# Endogenous conflict and the limits of predictive optimization

**DOI:** 10.1140/epjds/s13688-025-00599-x

**Published:** 2025-11-21

**Authors:** Thomas Chadefaux, Thomas Schincariol

**Affiliations:** Trinity College Dublin, College Green, Dublin, Ireland

**Keywords:** Conflict forecasting, Autoregression, Predictive modeling, Political violence, Temporal dependence

## Abstract

Forecasting models in political violence research increasingly rely on high-dimensional covariates and machine learning. Yet in practice, the most reliable conflict forecasts often come from much simpler systems: autoregressive models that predict future events based solely on recent past outcomes. This paper argues that such models are not merely convenient baselines but theoretically appropriate tools for sparse, dynamic environments like armed conflict. We show that autoregressive models consistently outperform or match more complex alternatives across multiple countries and specifications, while structural covariates frequently add little or degrade performance. We explain this pattern both theoretically and empirically: conflict is driven by internal feedback, burstiness, and short-term adaptation—not by slow-changing structural conditions. By foregrounding the limits of causal modeling in high-entropy settings, we make a broader case for epistemic modesty in prediction. Autoregression, we argue, is not a shortcut, but a principled strategy in systems that resist control.

Forecasting conflict is a hard problem. The data are noisy, the events are rare, and the systems that produce violence are complex, reactive, and only partially observable. Despite increasing access to structured data and sophisticated modeling techniques, predictive accuracy remains modest, and actionable foresight elusive [6, 18, 36]. A common response has been to expand the search for explanatory variables: political regime type, economic indicators, ethnic composition, environmental shocks [14, 16, 19]. The assumption is that with enough theory and covariates, we can capture the conditions under which violence emerges.

In many operational settings, however, the most accurate forecasts come from autoregressive models—systems that rely solely on a country’s own recent conflict history. These models ignore structural inputs, make no claim to causal insight, and simply project forward from the near past. Across a wide range of contexts and specifications, they consistently outperform or match more elaborate models that use political, economic, or demographic features.

This paper argues that this is not a statistical fluke or a modeling failure. In volatile, high-entropy systems like conflict, the recent past carries more predictive weight than slow-moving structural covariates. Conflict recurs because it escalates, persists, and fades through internal dynamics: retaliation, repression, mobilization, restraint. These mechanisms generate temporal dependencies that are directly encoded in outcome lags, but only indirectly, if at all, in conventional covariates. From a forecasting perspective, autoregression aligns more closely with how violence actually behaves.

We make this argument empirically and theoretically. Using monthly conflict data at the country level, we compare the performance of autoregressive models to models that combine lags with standard structural features. In nearly all cases, the lag-only models perform as well or better. We also provide a formal and intuitive rationale for this pattern: in sparse, reactive systems, the information required for short-term prediction is often endogenous to the system itself. External features, while useful for explanation, are often too slow, noisy, or unstable to improve forecasts.

More generally, autoregressive models offer a robust and transparent foundation for forecasting in systems where causal understanding is incomplete and intervention is risky. Rather than treating autoregression as a baseline to be outperformed, we propose treating it as a principled modeling strategy, one that is better suited to the limits of prediction in complex social systems.

## Why conflict dynamics are fundamentally autoregressive

Conflict systems tend to exhibit strong temporal dependence because they are driven by feedback, adaptation, and short-lived shocks. In this section, we argue that these dynamics are not anomalies or artifacts of modeling; they are features of how violence unfolds. As such, we should expect the near future to be predictable from the recent past, and we should expect models that ignore this structure to underperform.

### Conflict as a feedback process

Most theories of conflict emphasize that violence alters the conditions under which future violence occurs. A protest one week may lead to repression the next. An insurgent attack may trigger a retaliatory strike, which provokes further violence in turn. These feedback loops—escalation, retaliation, tit-for-tat dynamics—create endogenous momentum. This logic appears across scales and domains: in civil wars, urban unrest, electoral violence, and intergroup conflict.

The concept of the “conflict trap” formalizes this insight: countries that have experienced conflict face persistently elevated risk of recurrence due to weakened institutions, displaced populations, and normalized violence [12]. Tilly’s work on contentious politics highlights how protest and repression spiral together [34]. Kalyvas [22] documents how violence in civil wars often spreads locally through revenge, opportunism, and imitation. Studies of tit-for-tat retaliation demonstrate that actors respond to violence with violence, creating self-reinforcing cycles [15, 23]. Escalation dynamics are similarly well-documented: once conflict begins, interactions between adversaries tend to intensify rather than stabilize [1]. Even micro-level studies show that once violence occurs, actors quickly update expectations and incentives, often increasing the risk of recurrence [13].

### Event memory and operational rhythms

In addition to direct feedback mechanisms, conflict processes are influenced by a range of logistical and organizational constraints that inherently generate temporal dependencies, or autocorrelation, in conflict dynamics. Armed groups and state forces cannot act instantaneously; they require time to mobilize troops, plan operations, replenish supplies, and respond to evolving conditions on the ground. Supply lines must be reestablished, wounded personnel replaced, and actionable intelligence collected and analyzed before further action can be taken. These practical limitations introduce characteristic rhythms into conflict activity, often manifesting as bursts of violence followed by lulls, as well as delays in response and gradual decay in intensity. As a result, the immediate past becomes a valuable source of information for anticipating near-future developments, as recent events often signal continued momentum, logistical readiness, or reactive strategies in play.

Empirical work has shown that conflict events often exhibit clustering and semi-regular timing [3, 11]. Violence does not arrive in pure Poisson form; it arrives in waves. After a deadly clash, a temporary lull may follow as groups regroup. Then a new wave emerges. These rhythms may be driven by internal capacity, seasonal constraints, or strategic decisions [32]. Regardless of cause, they introduce serial dependence in the data.

Autoregressive models do not need to know the mechanisms behind this rhythm; they simply detect it. A spike in violence this month is often followed by elevated risk next month, and so on, often decaying gradually. Covariates cannot substitute for this memory. Even if structural features influence long-run trends, they do not encode short-run dynamics.

Furthermore, conflict actors are not static. They observe, adapt, and anticipate. Rebels may shift tactics in response to state patrols. Governments may change force posture in response to attacks. Civilians may flee, resist, or accommodate depending on the violence they have just witnessed. These responses are conditional on recent events—not just on deep structural features.

This introduces local learning and reactivity into the system. From a modeling perspective, it means the system is not exogenous: the behavior at $t+1$ is partially a response to behavior at *t*. In some cases, this adaptation is strategic (e.g., avoiding detection). In others, it is practical (e.g., pausing operations to regroup). Either way, the key point is that actors use history—and so should models.

Together, these dynamics suggest that autoregressive models are not crude approximations, but direct encodings of how conflict behaves. When a system has short-term memory, internal feedback, and temporal rhythms, a forecasting model that looks only at lagged outcomes can outperform more elaborate systems that assume stability in structural features.

This does not imply that covariates are useless. Structural factors may shape background risk or long-run trajectories. But if the goal is to anticipate near-term shifts—whether next month will be more violent than this one—then the best predictor is often the past. In this sense, the autoregressive model is not a baseline to be surpassed, but a benchmark that reflects the actual form of temporal dependence in the system.

### Autoregression and the limits of explanation

Forecasting and explanation are often conflated in social science. This has led to the assumption that predictive models should mirror causal theories—that variables which explain conflict across countries or decades will also help forecast its future course. In practice, this assumption frequently fails. Structural covariates such as regime type, GDP, or population may correlate with long-run conflict risk, but they rarely improve short-term forecasts. Theories that explain why some countries experience more violence than others often offer little guidance about what will happen next month in any given country.

This is not a failure of theory, but a feature of the forecasting problem. To make accurate predictions, a model must detect regularities that are stable over time, sufficiently strong to outweigh noise, and measurable at the relevant temporal resolution. In many dynamic systems, including conflict, these conditions are not met by structural variables. Such features tend to change slowly, are often poorly measured, and interact with local dynamics in nonlinear and context-specific ways. Even if they matter in a causal sense, they often lack the signal needed for short-term prediction.

Autoregressive models, by contrast, sidestep this problem. They make no assumptions about mechanisms or structure. Instead, they treat the system as a stochastic process with memory, learning directly from what the system has recently done. This may appear atheoretical, but in high-entropy settings where actors are reactive and feedback loops dominate, it is often the most appropriate modeling stance. The system’s recent behavior can encode more usable information than any static covariate.

To illustrate this point, consider a simple autoregressive process where the outcome $y_{t}$ is driven by its own immediate past:

1 $$ y_{t} = \rho y_{t-1} + \epsilon _{t}, $$

where $\rho \in (0,1)$ and $\epsilon _{t} \sim \mathcal{N}(0, \sigma ^{2})$. Here, the best predictor of $y_{t}$ is its own lag. This system exhibits inertia—recent values provide meaningful information about near-future outcomes. Suppose now we introduce a structural covariate $X_{t}$ that tracks long-run levels of *y* but is not responsive to recent variation:

2 $$ X_{t} = \mathbb{E}[y_{t}] + \eta _{t}, \quad \eta _{t} \sim \mathcal{N}(0, \sigma ^{2}_{x}). $$

This covariate may help explain variation across units, but it contains no information about within-unit transitions. A model that relies on $X_{t}$ alone will be unable to capture short-term dynamics, especially if $\eta _{t}$ is large or uncorrelated with $\epsilon _{t}$. This simple example reinforces three key points. First, explanatory variables are not automatically predictive. Second, even when causes are known, they may lack short-term forecast utility due to temporal mismatch. Third, autoregressive models, while simple, are often well-suited to the realities of dynamic systems like conflict, where near-future behavior is conditioned more by recent internal events than by external structure. In this sense, autoregression is not a fallback—it is an epistemically honest strategy for making forecasts when deeper causal understanding is limited, noisy, or strategically concealed.

## Revisiting modeling traditions in conflict forecasting

The study of conflict forecasting spans multiple disciplines and methodological traditions. Over the past two decades, the field has seen a shift from theory-driven, cross-sectional analysis to data-driven, high-resolution prediction. This section briefly reviews three major approaches to modeling conflict and situates our contribution in relation to them.

The earliest generation of conflict forecasting relied heavily on structural models derived from political science and economics. These models use country-level features such as GDP per capita, regime type, ethnic fractionalization, or geographic characteristics to estimate the likelihood of conflict onset [12, 14, 19]. The appeal of this approach lies in its theoretical grounding: structural variables are presumed to capture the root causes of political violence. However, structural models face several limitations. Many covariates are slow-changing and poorly suited for short-term prediction. Causal relationships may be unstable across contexts or time periods. Moreover, as Ward et al [36] and others have argued, these models often perform poorly in true out-of-sample settings, despite high in-sample fit. Their strength lies in explanation—not forecasting.

More recent efforts have adopted machine learning techniques, using large feature sets that include both structural and dynamic variables [20, 24, 28]. Projects such as ViEWS [21] and ICEWS [4] use gradient boosting, ensemble methods, or neural networks to forecast conflict across space and time. These models ingest event data, news reports, satellite imagery, and other real-time indicators to improve responsiveness and accuracy [25, 26]. While these approaches improve performance, they often obscure the source of the predictive signal. It is rarely clear whether improvements come from structural features, dynamic inputs, or sheer volume of data. In practice, many of these models derive much of their power from recent outcomes—effectively functioning as sophisticated autoregressive systems, even if not explicitly labeled as such. Our analysis clarifies and formalizes this point: much of what appears to be “complex modeling” is, in substance, a form of lagged outcome learning.

A smaller but growing line of work emphasizes temporal dependence as the central fact of conflict dynamics. Beck et al [2] demonstrated the importance of accounting for temporal dependence in international conflict studies. Walter [35] and Quinn et al [29] document strong patterns of conflict recurrence, showing that past violence is one of the strongest predictors of future violence. Brandt et al [5] argue for models that explicitly incorporate lagged outcomes, both to improve predictive accuracy and to better reflect the endogenous structure of violence. Our work extends this tradition by providing a broader theoretical justification for why autoregression should often be the default strategy—not just a technical baseline, but a theoretically grounded response to how conflict systems behave [8–10, 30, 31].

We offer three core contributions relative to existing work. First, we provide a clean empirical comparison showing that autoregressive models match or outperform structurally enriched alternatives across a wide set of forecasting tasks. Second, we offer a theoretical explanation for this pattern rooted in the endogenous and reactive nature of conflict. Third, we frame autoregression as not only a modeling choice, but a stance: a modest, epistemically cautious way to forecast behavior in systems that resist control.

## Data and forecasting task

To evaluate the performance of autoregressive forecasting in conflict systems, we implement a simple but representative forecasting task using country-level conflict data. Our goal is to assess how much predictive value is contained in the system’s own past, and whether standard structural covariates meaningfully improve forecasts.

We construct a dataset of monthly conflict fatalities and relevant covariates for a global sample of countries spanning 1989-2024. Our primary data source is the UCDP Georeferenced Event Dataset (GED) version 25.1 for conflict fatalities. Structural covariates are drawn from the World Bank World Development Indicators (GDP, population), Polity IV Project (regime scores), and standard cross-national datasets (ethnic fractionalization). The temporal coverage varies by variable, but all models are trained and evaluated on the 2018-2024 period to ensure consistent data availability. We then compare the predictive performance of two model classes: (1) autoregressive models that use only lagged outcomes, and (2) models that combine lagged outcomes with common structural covariates. Throughout, we focus on forecasting one month ahead.

Our target variable is the number of conflict-related fatalities in a given country-month. Fatalities are aggregated from the UCDP Georeferenced Event Dataset (GED) [33], which records individual violent events by date, location, and actor.^1^ We aggregate event-level data to the country-month level, summing total reported deaths due to organized violence. The data are log-transformed to reduce the effect of the skewness in the fatality distribution (see Table 2 and Fig. 10). This yields a time series $y_{c,t}$ for each country *c* and month *t*, where $y_{c,t}$ is a non-negative integer.

We focus on approximately 50 countries with sufficient conflict activity to allow informative forecasts. Specifically, we include countries with at least 50 months containing nonzero fatalities during the studied period. This threshold ensures adequate signal for model training and evaluation. We acknowledge that this selection criterion favors countries where autoregressive dynamics are likely to be detectable—precisely the setting where our theoretical claims should hold. However, this introduces an important limitation: our findings may not generalize to conflict onset in previously peaceful countries, where lags provide no signal. This is not merely a technical constraint but a substantive boundary condition on our argument. We return to this limitation in the Discussion section and note that onset prediction remains an important challenge for which hybrid approaches (combining AR and structural models) may be necessary.

We compare three model types:

- AR-only (AR): The outcome is modeled using its own lags. For each country-month, we include $y_{c,t-1}$ to $y_{c,t-6}$ as predictors, and a lagged conflict incidence (the previous year sum of fatalities).
- AR + Covariates (AR/Cov): We augment the AR model with common structural covariates, lagged by one month to avoid leakage.
- Covariates-only (Cov): A model using only structural features.

Structural covariates are selected from the PRIO-Grid and World Bank datasets to represent the most commonly used predictors in conflict forecasting literature. These include logged GDP per capita, the Polity IV regime score, logged population size, the ethnic fractionalization index, and lagged conflict incidence measured as a one-year sum. All covariates are lagged by one month. For robustness, we ensure that covariates are observed or interpolated prior to the forecast period.

We acknowledge that these are relatively coarse, slow-moving indicators, and that more fine-grained alternatives exist (e.g., monthly economic indicators, real-time political event data, or subnational measures). However, we deliberately chose these standard covariates for three reasons. First, they represent the variables most commonly invoked in structural theories of conflict and remain the basis for many operational forecasting systems. Second, if even these well-established predictors fail to improve upon autoregressive baselines, this strengthens our theoretical claim about the limits of structural modeling for short-term prediction. Third, these variables are consistently available across countries and time, ensuring replicability and comparability. That said, we recognize that our findings may be sensitive to covariate choice, and we discuss this limitation and potential extensions in the Discussion section.

The models are trained using a standard Random Forest (RF) approach. RF is an ensemble learning method based on decision trees that is robust to overfitting and well-suited for datasets with limited quantity and quality. Commonly used in conflict forecasting [21], this method offers strong predictive performance along with interpretability. It provides importance scores, which quantify the contribution of each variable to the model’s predictions. We performed hyperparameter optimization using grid search with time series cross-validation (3 splits) on data prior to 2018. Each model (AR, AR + Cov, and Cov) was tuned independently to account for their different feature spaces. The optimal hyperparameters were selected based on minimum root mean squared error (RMSE) across validation folds, ensuring no data leakage by maintaining strict temporal ordering in the cross-validation splits.

We apply a walk-forward (rolling origin) validation approach beginning in 2018. Specifically, the model is first trained on all available data up to December 2017. It is then used to generate one-month-ahead forecasts for each country-month in 2018 (January 2018, February 2018, …, December 2018). Crucially, for each forecast, we use *observed* values for the lagged variables—not predicted values. This means that when forecasting February 2018, we use the true observed fatalities from January 2018, and so on. This is standard practice in walk-forward validation and avoids compounding forecast errors.

After generating forecasts for all of 2018, the model is retrained on data through December 2018, and the same procedure is applied to generate forecasts for 2019. This process continues annually through 2024. Each year’s forecasts are generated using only information available up to the month immediately preceding the forecast.

We chose a one-month horizon (t + 1) for several reasons. First, it aligns with the temporal resolution at which autoregressive dynamics are strongest in conflict data. Second, it provides a clean test of the relative value of lags versus covariates without introducing additional forecast uncertainty from multi-step-ahead prediction. Third, while longer horizons may be valuable for some policy applications, one-month forecasts are operationally relevant for resource allocation, force posture adjustments, and humanitarian response planning. We also test longer-horizon forecasts (e.g., t + 3 or t + 6) and present the results in Appendix 2. We observe similar results at both horizons as at t + 1. We report two standard predictive metrics: Mean Absolute Error (MAE) and Mean Absolute Percentage Error (MAPE).^2^ Metrics are averaged across years and countries. The comparison between models is done by subtracting their respective MAE.

This forecasting task is intentionally modest. We do not fit deep models, or simulate interventions. Instead, we test a fundamental claim: that in conflict systems, the past contains most of what is forecastable, and that models which rely too heavily on external structural inputs may underperform not due to technical failure, but due to a mismatch between theory and signal.

**Figure 1:**
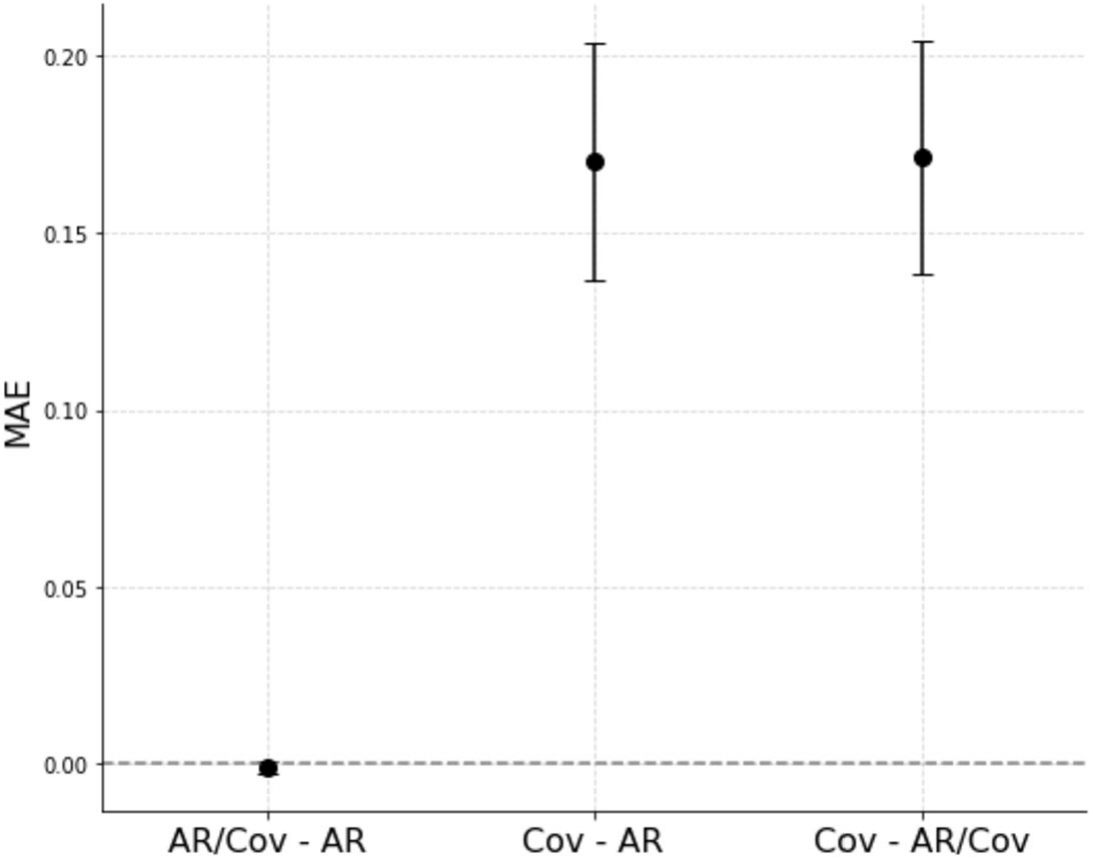
Difference in Mean Absolute Error (MAE) between the AR, AR/Cov, and Cov models. Points show the average difference in MAE between model pairs, with vertical lines representing the 95% confidence intervals. Positive values indicate that the model on the right outperforms the one on the left (lower error), and negative values indicate the opposite. Error bars show 95% confidence intervals computed from the standard error of the corresponding country–month absolute errors

Figure 1 shows that the purely autoregressive model (AR) and the covariate-augmented model (AR + Cov) perform similarly. To provide context for these comparisons, the absolute MAE values averaged across all countries and forecast periods are shown in Table 1. The inclusion of covariates to the AR model produced only a small, statistically insignificant improvement,^3^ representing a 0.2% reduction in error. Additionally, the inclusion of covariates even increases the relative error (MAPE) by 0.1%. In contrast, the model using only covariates (Cov) performs significantly worse than both models that include autoregressive components, with MAE and MAPE respectively 30% and 14% higher than AR, highlighting the critical role of autoregressive dynamics in predicting fatalities.

**Table 1:** Model comparison: mean scores with 95% CI (in parentheses) and relative differences vs AR

Metric	AR	AR + Cov	Cov
MAE	0.572 (0.019)	0.571 (0.019)	0.742 (0.022)
		−0.2% vs AR	+29.8% vs AR
MAPE	27.297 (1.378)	27.323 (1.382)	31.121 (1.329)
		+0.1%	+14.0%

**Figure 2:**
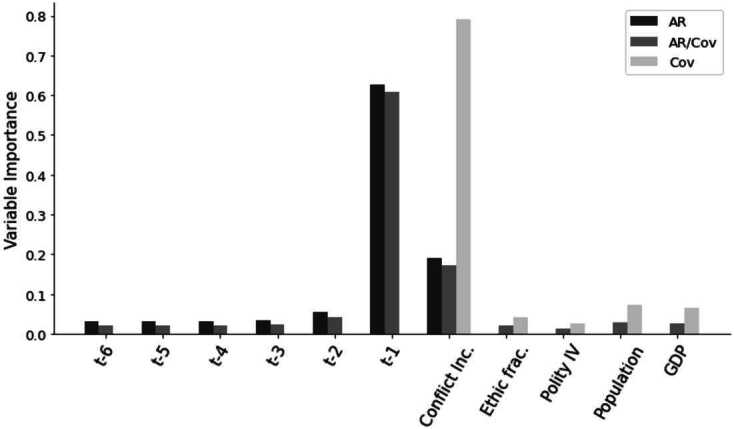
Importance Score for the AR model (dark grey), AR/Cov (grey) and Cov (light grey) variables. A higher score indicates a higher relative impact of the variable in the prediction

It is also visible through the importance score, presented in Fig. 2. The feature importance scores measure the average decrease in prediction accuracy when a feature is permuted across all trees in the ensemble, with higher scores indicating greater predictive contribution. The most important variables are the autoregressive ones, especially $t-1$ for AR and AR/Cov, and lagged conflict incidence for Cov. Conversely, the autoregressive variables after $t+2$ and the structural features have minimal importance.

### Stylized patterns of conflict recurrence

The strong performance of autoregressive models in our forecasting task is not accidental. It reflects structural regularities in how violence unfolds over time. In this section, we illustrate several stylized empirical facts about conflict dynamics that support the broader theoretical case: conflict is sparse, bursty, temporally dependent and most of the predictive signal lies in the recent past.

#### Sparsity and skew

**Figure 3:**
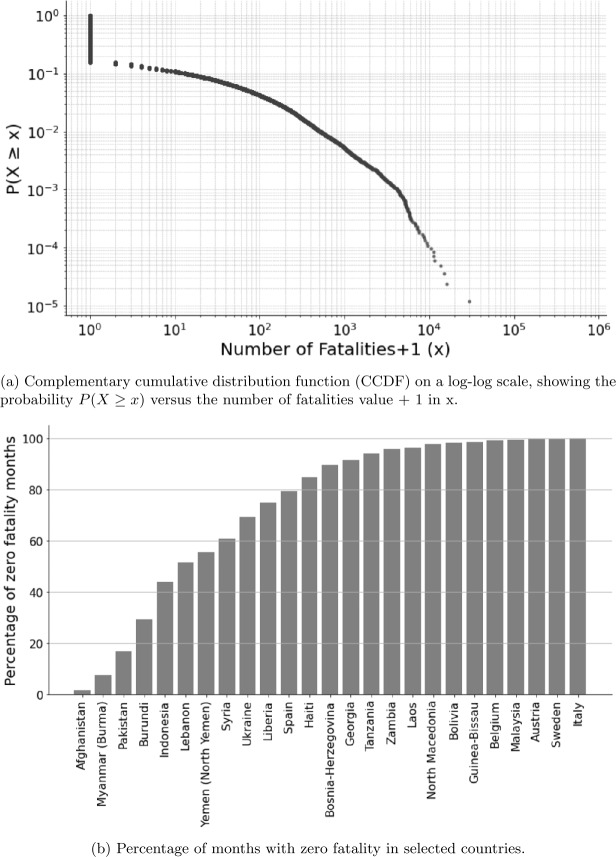
Empirical Cumulative Distribution Function (left) and some examples of percentage of zero value per country (right)

Conflict events are rare relative to the number of country-months in our dataset. Across the full sample, over 85% of country-months contain zero reported conflict fatalities (see Fig. 3a). The distribution of fatalities per country-month is highly right-skewed, with a long tail of high-casualty months. This sparsity means that any forecasting model faces a strong imbalance in the data, potentially leading to overall conservative predictions but also potential overpredictions when the model predicts more than zero fatalities.

Figure 3 clearly shows the rarity of conflict events. More than 80% of countries have more than half of zero values over the studied period. Forecasting these kinds of events requires a high degree of precision, and even small gains over a zero baseline constitute a nontrivial signal detection.

#### Autocorrelation and short-term memory

Despite sparsity, conflict events exhibit temporal clustering. If a country experiences violence in one month, it is significantly more likely to experience violence the next month. This pattern is visible in autocorrelation plots and in conditional probabilities.

**Figure 4:**
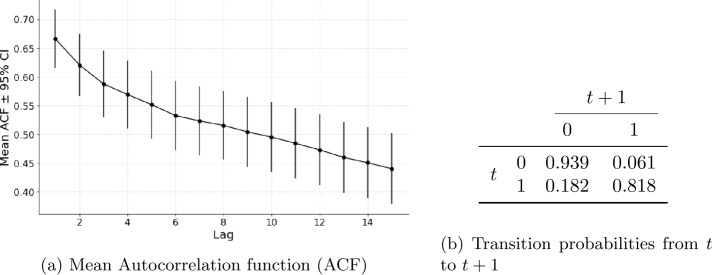
On the left, the average Autocorrelation function (ACF) across countries is plotted. The points represent the average value for each lag with their 95% Confidence interval. On the right, transition probability from *t* to $t+1$ using two simple representative classes: zero value (class 0) and positive value (class 1). The values are extracted using the selected country in the forecasting task. Error bars show 95% confidence intervals computed from the cross-country standard error of the country-level ACF values at that lag

Figure 4 presents the autocorrelation structure of the monthly fatality data, combining the average Autocorrelation Function (ACF) plot (left) with simplified class transition probabilities (right). The data have a short-term memory, indicated by a strong autocorrelation at low lags. When fatalities are categorized into binary classes, zero (class 0) and positive (class 1), the probability of transitioning between classes, particularly from 0 to 1, is low. These two indicators suggest that recent violence significantly increases the short-term risk of further fatalities, a dynamic that autoregressive models are well-suited to capture.

#### Burstiness and clustering

**Figure 5:**
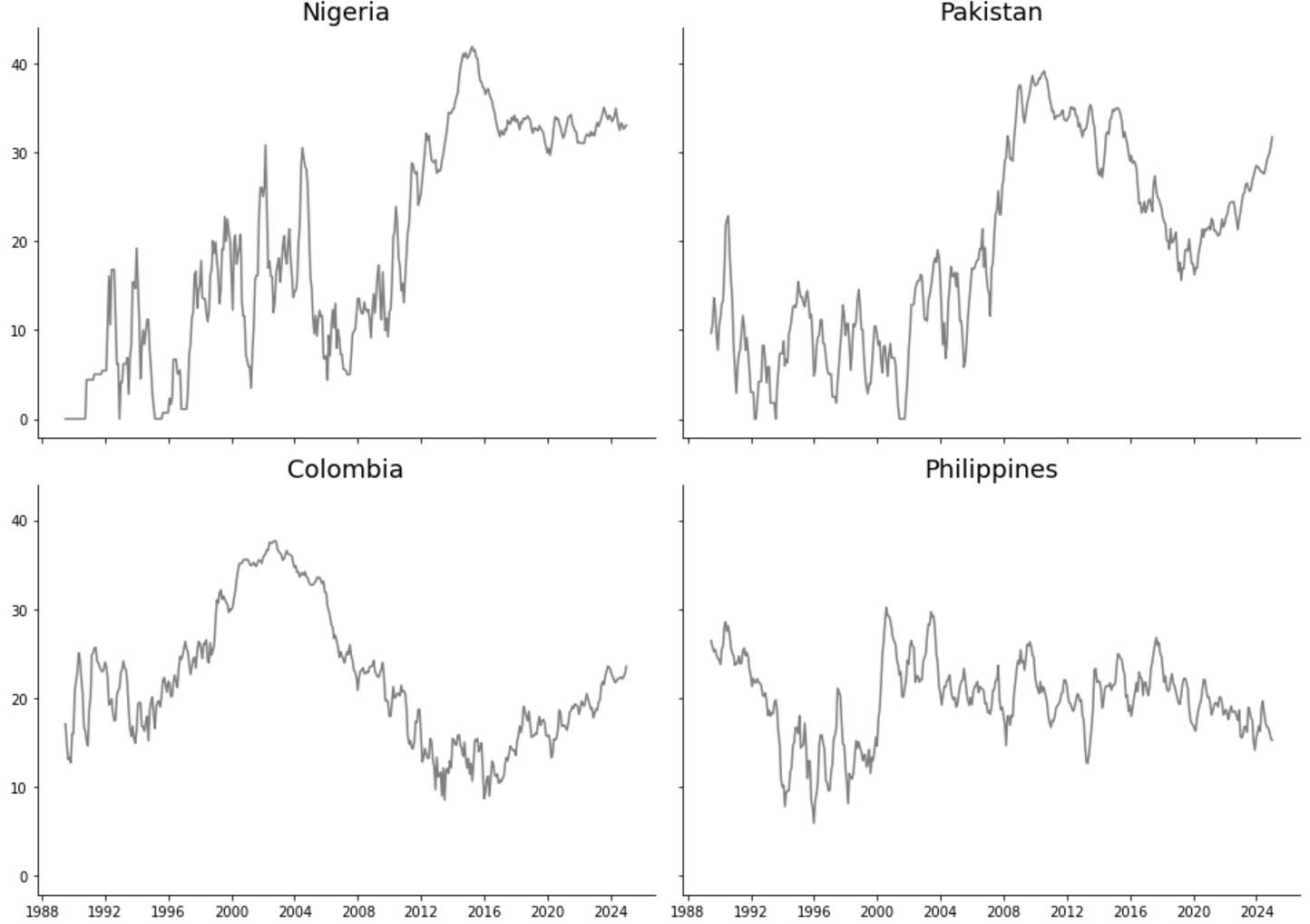
Rolling sum of fatalities over 6-month windows for 4 countries (Nigeria, Pakistan, Colombia, Philippines). Nigeria’s 2014–2015 spike tracks Boko Haram’s escalation, while Pakistan’s repeated surges align with intensified operations in FATA/KPK. Both cases illustrate the clustered bursts and gradual decay that autoregressive models capture

**Figure 10:**
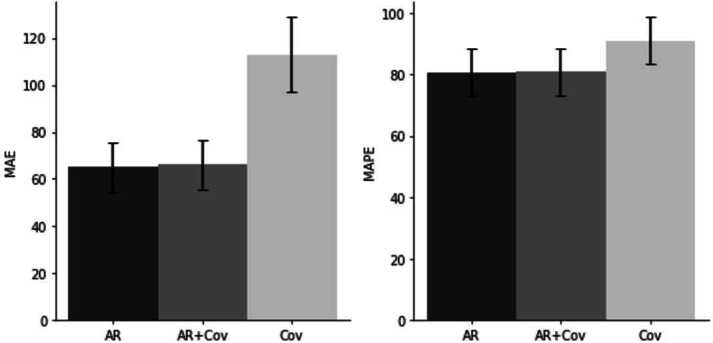
Model error with unlogged outcome. Bars show the average value of the model, with vertical lines representing the 95% confidence intervals

In many countries, violence does not occur at regular intervals. Instead, it occurs in short bursts—clusters of high activity followed by longer lulls. Burstiness refers to the tendency for events to arrive in concentrated periods rather than evenly distributed over time [17]. In time series terms, a bursty process exhibits variance in inter-event times that exceeds what would be expected under a Poisson (memoryless) process. Clustering refers to the spatial or temporal concentration of events such that occurrence in one period significantly raises the probability of occurrence in nearby periods. Figure 5 illustrates this pattern using rolling 6-month fatality sums for selected countries. These bursts often span 2–5 months, with local escalation followed by gradual decay.

These rapid shifts likely reflect local dynamics such as retaliation, suppression, or logistical constraints; factors that are not captured by slower-moving structural variables. Even with perfect measurement, structural features are unlikely to contribute meaningfully to short-term forecasting, though they may be more relevant for capturing long-term, annual trends. In contrast, autoregressive models, which rely on lagged outcomes, are better suited to capturing these fast-moving dynamics (see Appendices 2 and 3 for robustness tests).

These stylized patterns explain why autoregressive models perform so well in our setting. Conflict systems are not random, but neither are they purely structural. Their key behaviors—escalation, decay, persistence—unfold across time in locally observable ways. Models that draw directly on this temporal structure can detect signal that structural features miss. The result is a form of forecasting that is both statistically effective and theoretically aligned with the generative logic of conflict.

## Discussion

Our empirical results show that autoregressive models—those using only lagged observations of past violence—perform as well as, and sometimes better than, models augmented with structural covariates. This pattern is consistent across multiple countries, outcome types, and evaluation metrics. While some covariates occasionally improve predictions, the gains are inconsistent and modest. In many cases, they introduce noise or degrade performance.

This section interprets those results. We argue that the findings reflect deeper theoretical and epistemological features of conflict systems—namely, that they are driven more by internal dynamics than by stable external structures. We also reflect on the practical, philosophical, and ethical implications of this modeling perspective.

### Why do covariates fail to add value?

The disappointing performance of structural covariates is not a statistical fluke. It follows directly from their nature and function. Most structural features used in conflict forecasting—GDP, polity score, population, ethnic composition—are slow-moving, weakly correlated with short-term dynamics, and often inconsistently measured. They may reflect background risk, but they do not encode timing.

Forecasting, by contrast, is a temporal task. It requires estimating whether violence will increase, decrease, or recur in the near future. For this, the most relevant information is typically the recent past: the last month of fatalities, the last two months of protest, the last military confrontation. Structural covariates are simply not designed to carry this signal. Their failure is not due to poor modeling, but to a mismatch between what they represent and what forecasting requires.

Moreover, the temporal resolution of many covariates is coarse. Polity scores change annually, if at all. GDP data is updated yearly and often interpolated. Even when these measures are updated monthly, they do not reflect the short-run behavioral shifts that drive conflict escalation. In contrast, event data, even with reporting limitations, captures immediate behavioral changes. Autoregressive models use this signal directly, and thus better match the forecasting task.

### New data, same limits? Emerging covariates and the forecasting challenge

While our analysis focuses on traditional structural covariates, recent efforts in conflict forecasting increasingly turn to new data sources—news reports [7], Wikipedia edits [27], Earth observation, and social media [37]—in the hope of capturing more timely and granular signals. These data streams offer clear advantages. News and open-source intelligence are widely accessible and can track a wide variety of event types. Satellite imagery provides consistent coverage across space and time. Social media platforms, in some regions, capture local unrest that is not reported elsewhere.

But these sources face many of the same issues that plague conventional covariates—and introduce new ones. News and Wikipedia data suffer from temporal ambiguity: events are often reported after the fact, making it difficult to know whether the information is predictive or retrospective. They are also highly noisy and spatially imbalanced, reflecting geopolitical attention more than ground truth. Earth observation offers better spatial coverage but tends to focus on slow-moving ecological variables—climate trends, land use, nighttime lights—which may be poorly aligned with near-term conflict dynamics. Social media may capture otherwise invisible activity, but it is unevenly available, especially in rural or authoritarian settings, and shares the same issues of timing and noise.

In short, these sources are not exempt from the core constraints we identify. They may increase coverage or resolution, but they do not overcome the epistemic challenge of modeling reactive, endogenous systems. The fundamental issue remains: most covariates, old or new, struggle to add forecast value because they fail to capture the internal rhythms of conflict.

That said, these new streams are not without promise. They could enhance forecasting if used cautiously—perhaps not as direct predictors, but as context layers, regime shift detectors, or uncertainty signals. Our critique applies most strongly to static, structural covariates. A more optimistic reading is that autoregression sets a performance bar: new inputs must beat the past to be useful. As sensing technologies and event coding improve, the boundary between signal and noise may shift. For now, however, the case for epistemic modesty—and the strength of outcome-based modeling—still holds.

### Internal dynamics, not external structure

Conflict systems are primarily driven by internal dynamics. Violence generates responses—repression, retaliation, displacement—that feed back into the system. Tactical decisions, strategic adaptation, and emotional responses to trauma unfold over weeks and months, not decades. These are endogenous processes, structured more by what just happened than by underlying economic conditions. In complexity terms, conflict is a high-entropy, path-dependent system. Its trajectory is shaped by recent history, contingent shocks, and local actor interactions. This makes it resistant to structural modeling. The causes of violence are not fixed parameters; they are themselves dynamic, reactive, and embedded in the unfolding process.

Autoregressive models, by design, capture this kind of structure. They treat the past not as a nuisance to be controlled for, but as a signal to be learned from. They extract local regularities and short-term dependencies without assuming global rules or stable causal mechanisms. This makes them better suited to modeling systems that are unstable, adaptive, and historically contingent, all of which describe violent conflict.

### The practical case for time-based features

From a practical standpoint, time-based models offer several advantages. First, they are data-complete. Every country has a recent history. Even where structural covariates are missing, unreliable, or contested, the event record is usually available, especially for fatalities and major incidents.

Second, they are robust. Autoregressive models do not rely on the quality or comparability of GDP estimates, regime classifications, or survey-based indices. They reduce the number of modeling decisions and avoid the noise introduced by sparsely populated covariate spaces.

Third, they are generalizable. A model that uses only past outcomes can be trained and deployed consistently across countries and time periods. There is no need to recalibrate for each country’s unique data landscape. This makes AR models not only more accurate in many cases, but also more portable and scalable for policy use.

Autoregressive modeling also carries a philosophical message. It embraces what might be called epistemic modesty. Rather than attempting to model the world as it “should” behave based on theory, it models the world as it does behave based on observation. It resists the temptation to impose causal narratives on systems where causality is ambiguous or unstable.

This is particularly important in high-stakes domains like conflict, where the cost of false certainty is high. Covariate-based models often carry implicit causal claims: that democracy prevents war, that GDP reduces unrest, that ethnic exclusion drives violence. But these claims are contingent, context-dependent, and rarely reliable for forecasting. Using them as predictive levers can give a false sense of control. Autoregression avoids this problem. It does not speculate on why violence happens. It tracks how it moves. In doing so, it aligns the structure of the model with the structure of the system. It produces forecasts that are honest about what can be known, and silent about what cannot.

Forecasts are not neutral. They inform resource allocation, humanitarian deployments, early warning systems, and even military decisions. A model that overfits to covariates or amplifies speculative theories can distort these decisions. Worse, it can justify intervention based on fragile assumptions—for example that certain regimes are inherently unstable, or that specific populations are inherently prone to violence. Autoregressive models are less prone to this kind of overreach. They offer forecasts grounded in recent behavior, not theoretical bias. They reflect what has happened, not what should have happened. This makes them more cautious tools, but in high-risk policy contexts, caution is often a virtue.

### Forecasting as anti-optimization

Most predictive modeling assumes that better forecasts lead to better decisions. In stable domains, this assumption is often true. But in high-stakes social systems, such as violent conflict, the relationship between forecasting, intervention, and system behavior is more fragile. Attempts to optimize policy responses based on model outputs can destabilize the very dynamics being modeled. In these settings, more aggressive modeling does not guarantee better outcomes. In fact, it may do harm.

Conflict systems involve strategic, adaptive actors. These actors learn, react, and sometimes manipulate the informational environment. When forecasts are used for early warning, resource allocation, or targeted intervention, they become part of the system’s feedback loop. Rebels may shift location to avoid detection; states may intensify force to pre-empt predicted unrest; aid agencies may focus on hotspots, drawing attention or resentment. In such cases, the model is no longer an observer, it is a participant. Actors adapt to avoid being modeled, exploit blind spots, or reshape incentives. A forecast that was accurate ex ante becomes misleading once it drives action. Goodhart’s Law applies: when a measure becomes a target, it ceases to be a good measure.

This risk applies to *all* forecasting models, including autoregressive ones. However, AR models may be comparatively less vulnerable for two reasons. First, they do not rely on manipulable or strategically observable inputs. Structural covariates like GDP, regime scores, or ethnic composition can be politically contested, selectively reported, or influenced by policy. Recent conflict outcomes, by contrast, are harder to conceal or manipulate systematically, especially when aggregated from multiple sources. Second, AR models offer fewer apparent levers for targeted intervention. A forecast based on GDP or regime type suggests specific policy actions (economic aid, democratization); a forecast based solely on recent violence offers less actionable guidance beyond general preparedness. This makes AR forecasts less likely to trigger the kind of strategic adaptation that undermines model validity.

That said, AR models are not immune. If actors believe violence itself will trigger intervention, they may suppress reporting, stage events, or modulate intensity. The key difference is one of degree: AR models track behavioral outcomes that are harder to fully obscure, while structural models track indicators that are more subject to strategic manipulation. In this sense, AR models embody what we might call an *anti-optimization* stance—a modeling philosophy that prioritizes stability and minimal disruption over control and intervention.

This stance has ethical implications. Models that rest on fragile causal assumptions may appear powerful but carry outsized risks. When deployed, they can overfit to theory, amplify biases, or justify overreach. By contrast, lag-based models are more transparent and more cautious. They reflect recent behavior without attributing motive or projecting intervention logic. This makes them better aligned with principles of epistemic humility and policy restraint, particularly in settings where predictive error can escalate conflict, distort aid, or legitimize coercion.

### Limits and objections

While autoregressive models perform well in many forecasting settings, they are not without limitations. Below we address several common concerns, both conceptual and practical.

#### “Autoregression is tautological”

A common critique is that autoregressive models merely memorize the past. But this misunderstands their purpose. In forecasting, the relevant question is not whether a model explains the deep causes of conflict, but whether it can anticipate future states given available data. If past outcomes contain predictive signal—due to momentum, feedback, or actor behavior—then it is appropriate to exploit that structure. All forecasting relies on learned regularities; the virtue of AR models is that they do so transparently.

#### “What about interpretation and causal inference?”

Structural covariates often appeal to researchers and policymakers because they are interpretable and causally suggestive. But interpretability is not the same as predictive value. In many volatile systems, structural features are weakly correlated with short-term variation, even if they matter for long-run trends. Moreover, causality and forecasting are different goals. Our claim is not that structural factors are irrelevant to understanding conflict, but that their short-term predictive utility is often limited. In high-risk operational settings, actionable forecasts may require prioritizing reliability over explanation.

#### Do autoregressive models fail under regime change?

Yes, and so do most models. Like all statistical systems, AR models assume some degree of continuity. They perform poorly when the underlying data-generating process changes abruptly or enters a new regime (e.g., state collapse, sudden war onset). However, this is not a unique weakness of AR models. In fact, AR models may outperform other models here too: because they track the recent past, they are more likely to detect change as it begins. Structural models, by contrast, often impose fixed assumptions that lag behind reality.

#### Could more granular covariates improve performance?

Our analysis uses standard structural covariates (GDP, polity, population, ethnic fractionalization) that are commonly employed in conflict forecasting. These variables are relatively coarse and slow-moving, which may limit their utility for monthly predictions. More fine-grained alternatives—such as monthly economic indicators, real-time political event data, food prices, or subnational measures—might perform better. However, even if such variables improve forecasts, this would not undermine our core argument: that short-term conflict dynamics are primarily endogenous and that models must incorporate temporal dependence to be effective. Any improvement from finer-grained covariates would likely be additive to, not substitutive for, autoregressive structure. Future work should systematically test whether newer data sources (e.g., satellite imagery, social media, news-based measures) can outperform simple lags.

#### “Can they detect novel risks or first onsets?”

Autoregressive models are weakest when forecasting the onset of new conflicts in previously peaceful regions. In these cases, lagged outcomes provide no signal, and other methods may be necessary. However, first-onset conflict is rare in most datasets, and structural covariates have shown limited performance even in this domain [36]. One path forward is hybrid modeling: using AR methods for short-term dynamics and structural models for low-frequency change.

Autoregressive models are not all-purpose tools. They do not offer causal explanation, and they are less helpful when forecasting novel or extreme events. But within their intended use case—short-term prediction in systems with endogenous feedback—they offer a pragmatic and theoretically coherent approach. Their limits are real, but their strengths are well-matched to the challenges of forecasting in high-entropy environments.

## Conclusion

Autoregressive forecasting is often treated as a baseline—a tool to be outperformed by richer, more complex models. This paper has argued the opposite: in many conflict settings, autoregression is not just the simplest option, but the most appropriate one. Its strength lies in its alignment with how conflict behaves. Systems marked by feedback, momentum, and short-term adaptation are best forecast by models that track their own history.

Empirically, we have shown that lag-based models match or exceed the performance of more elaborate alternatives that include common structural covariates. This holds across multiple countries, model specifications, and outcome types. The addition of covariates rarely improves accuracy and often introduces noise or fragility. This is not due to poor implementation, but to a mismatch between theoretical assumptions and empirical signal. Structural features are slow-moving and inconsistently measured; the past, by contrast, is always present.

These findings have broader implications. Many domains of forecasting—from migration to public health to financial risk—share features with conflict systems: sparse events, volatile dynamics, incomplete causal knowledge. In such settings, autoregression offers not only robustness, but epistemic modesty. It does not speculate on mechanisms. It learns from what the system has already revealed.

Future work can build on this foundation. Hybrid models may combine autoregression with structural features in more principled ways, using the former as signal and the latter as context. Regime-switching approaches could detect when the predictive dynamics themselves are shifting—when lags stop being useful, or when conflict enters a new phase. Uncertainty quantification can also be layered on, improving risk communication without overpromising on accuracy.

The broader lesson is not that complex modeling is futile, but that in many high-entropy systems, restraint is a strength. Forecasting should begin with what is knowable, and in conflict, the past remains the most honest guide to the near future.

## Figures and Tables

**Figure 6:**
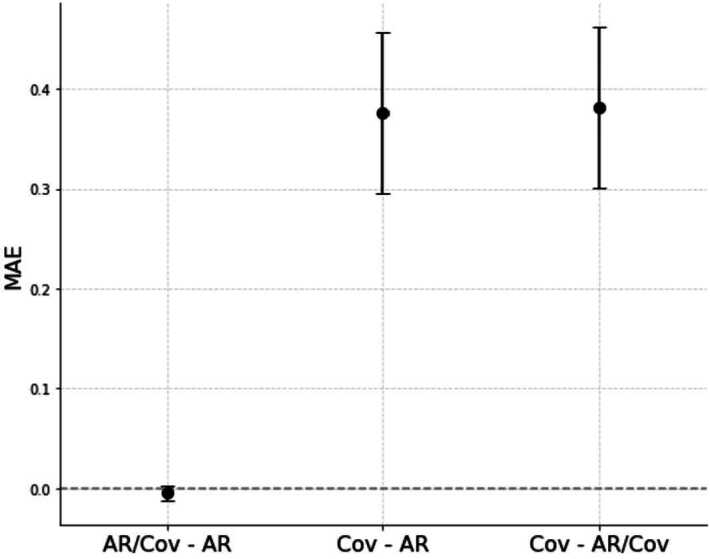
Replication of the Fig. 1 with the ACLED event dataset. Difference in Mean Absolute Error (MAE) between the AR, AR/Cov, and Cov models. Points show the average difference in MAE between model pairs, with vertical lines representing the 95% confidence intervals. Positive values indicate that the model on the right outperforms the one on the left (lower error), and negative values indicate the opposite

**Figure 7:**
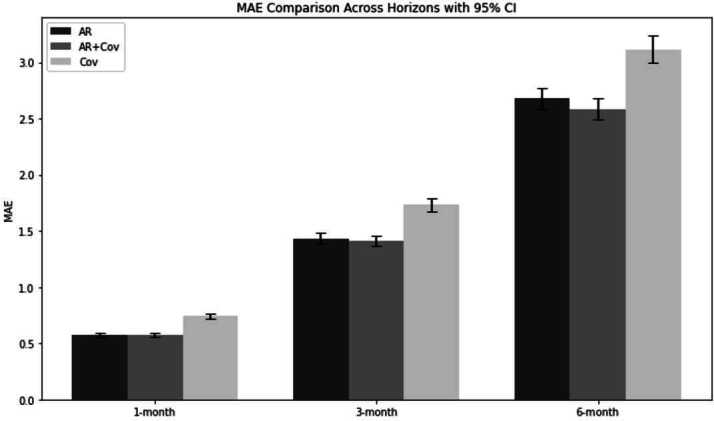
Mean Absolute Error of the models for the three different forecast horizons. Bars show the average error in MAE of models, with vertical lines representing the 95% confidence intervals

**Figure 8:**
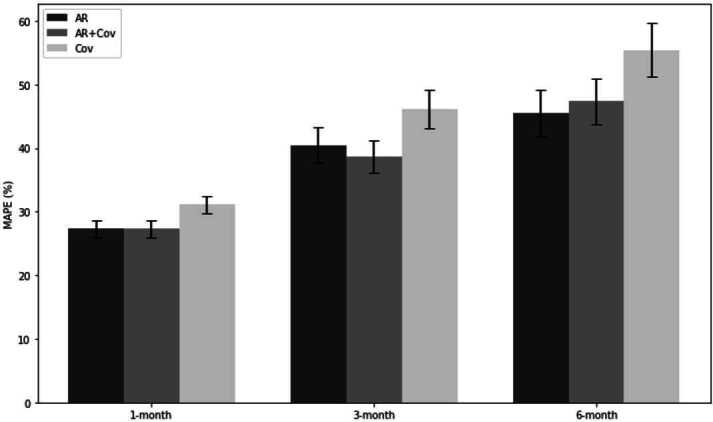
Mean Absolute Percentage Error of the models for the three different forecast horizons. Bars show the average error in MAPE of models, with vertical lines representing the 95% confidence intervals

**Figure 9:**
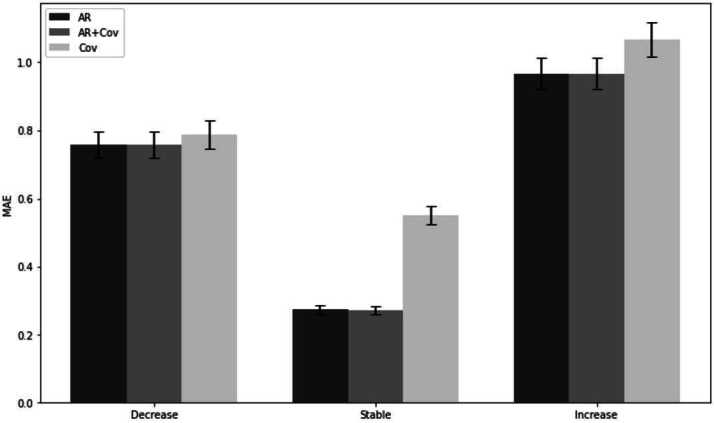
Model error aggregated by categories of percentage change. Bars show the average value of the model, with vertical lines representing the 95% confidence intervals

## Data Availability

Data from UCDP/ACLED used under their terms. All of the other material are owned by the authors and/or no permissions are required. Data and replication code is accessible via a publicly available github repository at https://github.com/ThomasSchinca/Limits_Conflict_Pred.

## Appendix 1: Robustness test with ACLED dataset

As a robustness test, we replicate the main test using the ACLED event dataset^4^ instead of the UCDP dataset. Similarly, we used the fatality number sum by country and month. The results of the test is presented in Fig. 6. The replication of the test gives similar results using ACLED event dataset: the AR and AR + Cov models produce significantly similar performances, and significantly worse for the Cov model.

## Appendix 2: Robustness test on forecast horizon

As a robustness test, we extend the forecasting task to horizon t + 3 and t + 6. The MAE is reported in Fig. 7 and the MAPE in Fig. 8.

The MAE of the AR model increases slightly as the forecast horizon extends, but the deterioration is much smaller than that of the Cov model. Additionally, while the MAPE at t + 3 is higher for the AR model than for the AR + Cov model, it becomes lower at t + 6. Consistent with the MAE results, the Cov model has the highest error across all forecast horizons.

## Appendix 3: Additional test on the dynamic

In this test, we aggregated the model’s error based on the percentage of change, calculated as $diff_{\%} = \frac{fat_{t} - fat_{t-1}}{fat_{t-1}}*100$. The errors are grouped into three categories: decrease (when the percentage is lower than −5%), stable (between −5% and 5%), and increase (higher than 5%). The results are shown in Fig. 9. As expected, the increase and decrease categories show higher error values. The AR and AR + Cov models consistently perform best across all three categories.

## Appendix 4: Additional figures

**Table 2:** Descriptive statistics for monthly conflict fatalities dataset

Statistic	Value
Countries	117
Country-Months	50,544
Zero-Fatality (%)	74.79
Mean (Raw)	57.76
Median (Raw)	0.00
Range (Raw)	0–630,417
Mean (Log)	0.87
Median (Log)	0.00
Range (Log)	0.0–13.354

## Footnotes

1 We replicate the analysis with the ACLED Dataset, included in Appendix 1.

2 Because MAPE is undefined when the true value is zero, observations with zero fatalities are excluded from its calculation.

3 One-sample t-test for difference from zero: p-value = 0.47.

## Abbreviations

AR: Autoregressive model
AR/Cov: Autoregressive model with structural covariates
Cov: Covariates-only model
GED: Georeferenced Event Dataset (UCDP)
GDP: Gross Domestic Product
PRIO: Peace Research Institute Oslo
RF: Random Forest (ensemble machine learning method)
MAE: Mean Absolute Error (forecast performance metric)
MAPE: Mean Absolute Percentage Error (forecast performance metric)
CCDF: Complementary Cumulative Distribution Function
